# Beyond the Edge of Hypomethylating Agents: Novel Combination Strategies for Older Adults with Advanced MDS and AML

**DOI:** 10.3390/cancers10060158

**Published:** 2018-05-24

**Authors:** Anne Sophie Kubasch, Uwe Platzbecker

**Affiliations:** 1 Medical Clinic and Policlinic I, University Hospital Carl Gustav Carus, TU Dresden, 01307 Dresden, Germany; annesophie.kubasch@ukdd.de; 2 National Center for Tumor Diseases (NCT), University Hospital Carl Gustav Carus, TU Dresden, 01307 Dresden, Germany; 3 German Cancer Consortium (DKTK), 01307 Dresden, Germany; 4 German Cancer Research Center (DKFZ), 69120 Heidelberg, Germany

**Keywords:** HMA, elderly AML, combination strategies, checkpoint inhibition

## Abstract

Higher-risk myelodysplastic syndrome (MDS) and acute myeloid leukemia (AML) of the elderly exhibit several commonalities, including first line treatment with hypomethylating agents (HMA) like azacitidine (AZA) or decitabine (DAC). Until today, response to treatment occurs in less than 50 percent of patients, and is often short-lived. Moreover, patients failing HMA have a dismal prognosis. Current developments include combinations of HMA with novel drugs targeting epigenetic or immunomodulatory pathways. Other efforts focus on the prevention of resistance to HMA using checkpoint inhibitors to enhance immune attack. This review focuses on recent advances in the field of HMA-based front-line therapies in elderly patients with myeloid diseases.

## 1. Introduction

At diagnosis, median age of patients with acute myeloid leukemia (AML) is 67 years, which means that more than half the cases are elderly adults [1,2]. The same is true for myelodysplastic syndromes (MDS), with a median age of approximately 70 years at diagnosis. Ongoing demographic changes will therefore result in an increased proportion of elderly patients, and consequently, the prevalence of MDS and AML will rise significantly [3]. The mostly dismal prognosis of these patients compared to younger adults is not only caused by additional comorbidities; the disease biology also differs [4], including a higher rate of secondary AML with prior MDS and treatment-related disorders [5,6]. These factors account for higher rates of early mortality, lower complete response, and high relapse rates even after intensive chemotherapy (ICT) or hematopoietic stem cell transplantation (HSCT) [7]. Interestingly, higher-risk (HR) MDS and AML of the elderly share common phenotypic and genetic characteristics, like overlapping somatic mutations in multiple components of the RNA splicing machinery (*SRSF2*, *U2AF1*, *SF3B1*) across WHO-defined blast thresholds. Studies have demonstrated that SF3B1-mutant HR-MDS and AML patients are clinically, cytologically, and molecularly highly similar; this overrides the artificial separation between both diseases [8]. Additionally, AML with secondary-type (MDS) mutations are more often found in older individuals, and display a median of four different mutations in myeloid driver genes per case, which is twice as many as the *de novo* AML type [9]. Moreover, mutations which are commonly mutated in MDS, including *SRSF2*, *SF3B1*, *U2AF1*, *ZRSR2*, *ASXL1*, *EZH2*, *BCOR* and *STAG2*, have been specifically linked to secondary AML as opposed to *de novo* AML patients, suggesting that they may primarily drive the specific biology of the disease [9].

Over the last years, treatment outcomes have improved in younger patients, but remain poor in older adults with HR-MDS or AML [10,11]. A recent study analyzed the therapeutic course and outcome of 976 patients treated by ICT between 2000 and 2014. In the 513 analyzed younger patients, the period of time was significantly associated with a better overall survival, especially during the years 2010–2014. Interestingly the same was true for older patients (*n* = 463) [12], although only a minority, especially beyond 70 years of age, is considered eligible for such therapy.

Treatment algorithms of older patients had remained essentially uniform for decades, but changed after the introduction of hypomethylating agents (HMAs) [13]. HMAs such as decitabine (DAC) or azacitidine (AZA) have shown to improve overall survival (OS) compared to conventional care options, including low-dose cytosine arabinoside (LDAC) and ICT in older patients [14,15]. Thus, until today, one of the important issues in older AML patients is the decision between ICT and HMA. However, responses in these patient cohorts are rather low, are often short-lived, and outcome after HMA failure is seldom favorable.

Thus, in this review, we will discuss upcoming novel treatment strategies beyond single agent HMAs for older patients unsuitable for standard ICT.

## 2. Hypomethylating Agents between Then and Now

### 2.1. Historical Development of Hypomethylating Agents in MDS and AML

AZA and DAC are both cytosine analogues developed in the 1960s [2]; however, the have different modes of action: DAC can be incorporated into DNA strands, while AZA acts by incorporating itself into both DNA and RNA chains. In 1993, the first phase II trial using 75 mg/m^2^ AZA for 7 days every 28 days, over 6 cycles involving 43 patients with HR-MDS had been published. In the trial, 49% of patients responded, and results have been very closely reproduced in subsequent phase II and III trials [16]. In 2009, a phase III, multicenter open-label trial (AZA-MDS-001) resulted in the approval of the use of AZA in patients withup to 30% bone marrow (BM) blasts. This pivotal trial included 358 patients with HR-MDS, randomly assigned to receive either AZA or conventional care regimens (CCR), like best supportive care (BSC), LDAC, or ICT, according to investigator’s choice [14]. Median overall survival was 24.5 months for the AZA treated patients, versus 15.0 months in the conventional care group. Thus, AZA significantly prolonged survival in patients with International Prognostic Scoring System (IPSS) intermediate-2 or HR-MDS, compared to conventional care, which led to FDA and EMA approval for MDS patients not eligible for allogeneic HSCT. Response rates comprised 49% of AZA-treated patients, including an improvement of blood counts (hematologic improvement (HI) according to IWG criteria), as well as 29% of patients with either complete remission (CR) or partial remission (PR) [14].

In 2011, low-dose DAC 15 mg/m^2^ IV over 4 h three times a day for 3 days, in 6-week cycles, was compared to BSC only in 233 HR-MDS patients older than 60 and not eligible for ICT, in a randomized phase III trial. There was no significant prolongation of median OS comparing DAC to BSC (10.1 versus 8.5 months, respectively), but progression-free survival (PFS) was significantly longer in patients treated with DAC compared to BSC (median PFS 6.6 versus 3.0 months, respectively) [17]. Response rates have been determined, with 13% of patients achieving CR, 6% PR, and 15% HI rates in the DAC arm. Thus, the trial confirmed activity of DAC in HR-MDS with an overall response rate (ORR) of 34%, including HI. Additional multivariate analyses indicated that the following features were of independent poor prognosis regarding OS, PFS, and progression into AML: IPSS high risk, poor cytogenetics, less than 3 months of MDS duration, and ECOG PS of 1 or 2 [17]. Also based on further studies, DAC was approved for MDS by FDA, but not by EMA, because of the mentioned absence of a clear survival benefit [18].

In 2012, DAC was evaluated in a multicenter randomized phase III trial in 485 older (≥65 years), newly diagnosed AML patients with poor or intermediate- risk cytogenetics compared to CCR like BSC or LDAC. A post-hoc analysis revealed a significant increased OS rate in favor of DAC (7.7 versus 5 months, respectively), including a better CR rate (17.8% versus 7.8%, respectively) [19]. In an exploratory subgroup analysis, treatment benefit from DAC was more clearly observed in patients ≥70 years, *de novo* versus secondary AML, baseline BM blasts above 30%, intermediate- versus poor-risk cytogenetics and ECOG PS of 2 versus 0 to 1 [18]. In fact, focusing on older AML patients with BM blast ≥30%, CR/CRi rate was 27% for patients receiving DAC versus 11% with CCR, which translated into a significant survival benefit for patients receiving DAC (median 8.6 vs. 4.7 months, respectively) [20]. Several single-arm studies have further confirmed the activity of DAC in older patients with AML. Subsequently, EMA, but not FDA, approved DAC for the treatment of adult patients aged ≥65 years with newly diagnosed, *de novo* or secondary AML, who were ineligible for treatment by standard ICT. 

In 2015, the AZA-AML-001 phase III trial, copying the trial design of the respective MDS study [14], investigated AZA versus CCR in 488 newly diagnosed AML patients (median age of 75 years) and more than 30% BM blasts. Overall response (CR/CRi) rates were comparable in the AZA (27.8%) and CCR (25.1%) arms [14]. AZA prolonged median survival compared to CCR by 3.9 months, and was more active across all subgroups, including patients with poor risk cytogenetics [20]. Data of this study resulted in extended EMA approval of AZA for the treatment of adult patients aged 65 years or older with AML and more than 30% of BM blasts who are not eligible for HSCT.

### 2.2. Predicting Response and Outcome with HMA Treatment 

HMAs are given continuously until progression and median response duration is about one year. However, a subset of patients has long lasting remission, in rare cases for more than 3–4 years [17,18]. Nevertheless, response to HMAs cannot be predicted, and seems to occur rather independently of clinical variables. However, several factors predict duration of response and survival, as shown by a large French study. The study group investigated 282 patients with intermediate-2, HR-MDS and AML with ≤30% marrow blasts, undergoing single agent AZA treatment. In this cohort, BM blast count < 15%, normal karyotype, and no previous treatment with LDAC independently predicted better response to AZA, while patients with complex karyotypes, red blood cell (RBC) transfusion dependence, poor ECOG performance status, and the presence of circulating blasts all independently predicted shorter response durations and OS. Those factors have been combined in a simple prognostic score defining 3 patient subsets with significantly different survival rates (low, intermediate, high). The study also confirmed that in patients failing to reach CR or PR, achievement of HI, notably of HI-E, was associated with improved OS after AZA treatment [21].

The European ALMA score (E-ALMA) categorized three risk groups with different survival and response rates to AZA (favorable, intermediate, unfavorable), based on ECOG, white blood cell counts (WBC) before AZA onset, and cytogenetics (normal or abnormal). AZA seems to be a reasonable treatment option, especially for patients with a favorable or intermediate E-ALMA scores [22]. Bores et al. hypothesized that the use of G-CSF might be associated with a better response to AZA, and that a low initial lymphocyte count has a negative impact on survival after AZA treatment [23]. 

Moreover, recent developments in the molecular characterization of MDS and AML have revealed the presence of the mutations involved in epigenetic processing (e.g., TET2, ASXL1, DNMT3A). The impact of different mutations on response to AZA in MDS or AML has been evaluated in several studies. One of the first studies sequenced the TET2 gene in 86 MDS and AML patients with up to 20–30% blasts treated with AZA. While 15% of these patients carried TET2 mutations, ORR including HI was 82% in TET2 mutated versus 45% in wild-type patients. Thus, mutated TET2 and favorable cytogenetic risk independently predicted a higher response rate to AZA treatment. However, response duration and OS were not different [24]. Bejar et al. sequenced 40 recurrently mutated genes associated with myeloid malignancies in 213 MDS patients (receiving either AZA or DAC). Again, TET2 mutations predicted a better response to HMA treatment, but only in the absence of ASXL1 mutations. On the other hand, mutations of TP53 and PTPN11 were associated with shorter OS [25]. Craddock et al. also evaluated the impact of mutational status on clinical response to AZA by analyzing 250 patients with newly diagnosed, relapsed, or refractory AML or HR-MDS. Lower CR (CR, CRi, mCR) rates occurred in patients with an IDH2 and STAG2 mutation, higher CR rates in patients with NPM1 mutation. Mutations in CDKN2A, IDH1, TP53, NPM1, and FLT3-ITD were associated with a worse OS in univariate analysis, while multivariate analysis showed a decrease in OS in patients with CDKN2A, IDH1, and TP53 mutations. Moreover, no mutations have been shown to be associated with improved OS, but ASXL1 and ETV6 have been demonstrated to be associated with reduced response time to AZA treatment [26]. 

Welch et al. investigated molecular determinants of clinical responses after a 10-day DAC regimen in 116 patients with AML, including 26 patients with MDS. The clearance of leukemia-specific mutations correlated closely with morphologic and cytogenetic responses. Especially mutations in TP53 and SF3B1 genes showed consistent, rapid reductions in variant allele frequency, to levels of less than 5% after DAC treatment. Surprisingly, all patients with TP53 mutations had a response after DAC treatment with BM blast clearance, as compared to 47% of patients without TP53 mutations [27]. In other studies evaluating the effect of TP53 mutation on ORR during HMA therapy, patients with TP53 mutation had rates of response that were similar, but not higher, compared to patients with wild-type TP53 [28,29]. 

Currently, there are no prospective data reporting on a direct comparison between AZA and DAC. However, retrospective data do not indicate a clear superiority of either compound [30].

### 2.3. Novel Hypomethylating Agents (HMA)

#### 2.3.1. Guadecitabine 

Guadecitabine, a dinucleotide of DAC and deoxyguanosine, is a new-generation DNA hypomethylating drug that shows similar potency, but improved pharmacokinetic properties. The agent was designed to prolong the exposure of tumor cells to its active metabolite DAC [1]. Guadecitabine is given in a small volume subcutaneously, resulting in a lower peak concentration level, a longer half-life because of its resistance to cytidine deaminase degradation, and extended exposure to its active metabolite compared with intravenous DAC [31]. Similar to other HMAs, responses to guadecitabine occur slowly, 20% of remissions occur after 6 cycles [31,32].

The first phase II study included 103 patients with relapsed/refractory AML; most of them had prior ICT. Patients were randomized into two cohorts: in the first cohort, patients received either 60 mg/m^2^ or 90 mg/m^2^ guadecitabine on days 1–5 (5-day regimen). The second cohort was treated with a 10-day regimen: 60 mg/m^2^ guadecitabine on days 1–5 and days 8–12, for up to 4 cycles, followed by 60 mg/m^2^ for 5 days in subsequent cycles. It was shown that 23% of all patients had CRc to guadecitabine, and the median survival was 6.6 months; 19% of patients were alive after 2 years [31]. In a second, randomized dose–response study, guadecitabine was given to 51 treatment-naïve, elderly patients with AML, who were not eligible for ICT, at 60 or 90 mg/m^2^ per day subcutaneously for 5 days in 28-day cycles. There were no significant differences between these two doses in overall CR rates of all treated patients [33]. A more intense, 10-day regimen for the first 1–2 cycles had similar CR rates. Currently, there is a phase III trial with 5-days guadecitabine at 60 mg/m^2^ per day subcutaneously versus the physician’s choice of treatment (cytarabin, DAC or AZA), in patients with previously untreated AML who are ineligible for ongoing treatment with ICT (NCT02348489, Table 1). 

Currently, guadecitabine compared to LDAC/AZA/DAC is being tested in a phase III trial (ASTRAL-1, NCT02348489) in 800 treatment naïve AML patients, who are ineligible for treatment with ICT. Guadecitabine is also under investigation in a phase III trial (ASTRAL-2, NCT02920008), including 404 AML patients who failed or relapsed following prior ICT.

In solid tumors, there are discussions about whether guadecitabine might sensitize tumor cells to immunotherapeutics, and resensitize resistant cancer cells to chemotherapeutic drugs [1,34]. 

#### 2.3.2. Oral Azacytidine 

Another exciting, “novel” HMA is an oral formulation of 5-azacitidine (CC-486), which may provide a more convenient means of administration than the conventional AZA SC [35]. Furthermore, given the longer exposure time, this might even improve the efficacy of AZA. As an epigenetic modifier and DNA methyltransferase inhibitor, the drug is currently in clinical development for treatment of hematologic malignancies. In an expanded phase I trial (NCT00528983), patients with IPSS LR-MDS and median patient age of 72 years received 300 mg CC-486 once daily for 14 or 21 days of repeated 28-day cycles. ORR have been determined with 36% of patients receiving 14-day dosing, and 41% for those receiving 21-day dosing, including HI in 28% of patients and RBC TI sustained for 56 days in 47% of patients with baseline transfusion dependence. Notably, rate of grade 3–4 neutropenia with CC-486 was lower (16%) than that reported for AZA SC and DAC (32% versus 29%, respectively) in this study [36]. 

Based on these results, the randomized, placebo-controlled phase III QUAZAR LR-MDS trial (AZA-MDS-003, NCT01566695) with a target enrollment of 386 LR-MDS patients with red blood cell transfusion depended anemia and thrombocytopenia was initiated, comparing 300 mg CC-486 for 21 days of repeated 28-day cycles plus BSC versus placebo plus BSC. 

Low-dose CC-486 (200 or 300 mg for the first 7 or 14 days of each 28-day cycle) is also currently being investigated in a phase I/II trial (CC-486-AML-002, NCT01835587) examining its use as a maintenance therapy following HSCT in ~30 patients with MDS or AML (QUAZAR Post-Transplant Study). Additionally, a phase III study is ongoing, where oral AZA (300 mg for 14 days of 28-day cycles) is evaluated as a maintenance therapy after ICT without transplant in AML patients (QUAZAR AML Maintenance study, CC-486-AML-001, NCT01757535). 

## 3. Novel Combination Strategies with HMA

### 3.1. Venetoclax 

BCL-2 is a pro-survival protein preventing apoptotic cell death and facilitating MYC-induced transformation [37]. The protein is also overexpressed in hematologic malignancies, where it has been implicated in therapeutic resistance of AML cells [38]. Prior studies demonstrated that BCL-2 inhibition reduces oxidative phosphorylation, leading to eradication of quiescent leukemic stem cells [39]. Venetoclax (VEN) acts as an orally selective BCL-2 inhibitor. In 2017, FDA approved VEN for treatment-naïve AML patients at least 65 years of age, in combination with LDAC. The associated phase I/II study is still ongoing, preliminary data presented an ORR of 61% and a CR rate of 21% in older, induction-ineligible patients [40]. The administration of single-agent VEN in relapsed AML has resulted in only modest responses; a single-arm phase II study, including 32 patients with relapsed or refractory AML, demonstrated an ORR of about 19% [41]. More promising are novel approaches, evaluating the combination of VEN with DAC or AZA in treatment-naïve AML patients ≥65 years, with intermediate or poor-risk karyotype, and who are ineligible for standard induction therapy. In this trial, 145 patients received DAC (Arm A: 20 mg/m^2^ IV) daily, on days 1−5, or AZA (Arm B: 75 mg/m^2^; SC or IV) daily, on days 1−7 of each 28-day cycle, in combination with once-daily oral VEN (400 mg or 800 mg). Preliminary data shows 41% CR in patients with intermediate- and 30% CR in patients with poor-risk cytogenetics. Median OS in all patients was 17.5 months. A subgroup analysis comparing AZA to DAC, and VEN 400 mg versus 800 mg is currently ongoing. Using VEN, the most common treatment emergent adverse events were nausea and febrile neutropenia [42]. 

Another phase Ib/II open-label study (NCT02287233) evaluated VEN combined with LDAC in patients ≥65 years and previously untreated AML, who were ineligible for treatment with ICT. Among patients given VEN 600 mg and LDAC, 62% achieved CR/Cri (26% CR, 36% CRi, and 2% PR), with a median duration of CR/CRi of 14.9 months [43]. In the salvage setting, 43 patients with a median age of 68 years, and relapsed/refractory AML or MDS, received VEN in combination with HMA or LDAC. ORR was 21% and median survival was 3.0 months. Responses occurred in 24% of patients with intermediate-risk cytogenetics, and in 27% of patients with IDH1/2. In the same study, responses were recorded in 50% of patients with RUNX1 and 20% with TP53 mutation [44].

### 3.2. Lenalidomide 

Lenalidomide (LEN) belongs to a class of compounds called immunomodulatory derivatives (IMiDs). LEN has been shown to possess anti-angiogenic activity through the inhibition of basic fibroblast growth factor (bFGF), vascular endothelial growth factor (VEGF), and TNF-alpha induced endothelial cell migration [45]. In addition, LEN has a variety of immunomodulatory effects, stimulating T cell proliferation and the production of various cytokines [46,47]. Casein kinase 1A1 (CK1α) is encoded by a gene within the common deleted region for del(5q) MDS. It has been shown that LEN induces the ubiquitination of CK1α, resulting in CK1α degradation, providing an important basis for the unique mode of action of LEN in these patients [48].

A phase III randomized study evaluated LEN in 205 IPSS low-/intermediate-1-risk del5q MDS patients with RBC transfusion-dependency. Patients received a placebo or LEN 10 mg/day on days 1–21, or 5 mg/day on days 1–28, of a 28-day cycle. More patients in both LEN arms achieved RBC-transfusion independence (TI) compared to patients receiving a placebo (56.1% and 42.6% versus 5.9%). After LEN 10 mg/day, cytogenetic response rates were 50.0% versus 25.0% after LEN 5 mg/day [49]. As a result, the drug was approved by FDA in 2005, specifically for the treatment of LR-MDS patients with del(5q) and transfusion dependency. 

In other clinical trials, LEN monotherapy has been evaluated extensively in previously untreated, older (>60 years) AML patients, with or without del(5q). In one of these studies, AML patients with del(5q) were treated with LEN 50 mg daily for 28 days as induction therapy, and 10 mg daily for 21 days of a 28-day cycle, as maintenance therapy. Among the 37 evaluable patients, 14% achieved a PR or CR, with relapse-free survival of about 5 months. Median OS was 2 months for the entire population, showing a modest activity of LEN monotherapy in older del(5q) AML patients [50]. 

Another study evaluated 33 newly diagnosed AML patients ≥60 years, without isolated 5q abnormalities. Again, patients received LEN at 50 mg daily for up to two 28-day cycles, and 10 mg LEN daily as maintenance therapy. After LEN induction therapy, the overall CR/CRi rates were 30% and 53% respectively, albeit with a median OS from study enrollment of about 4 months only [51].

The combination of AZA and LEN is supported by reasonable single-agent activities across all stages of MDS and AML, irrespective of their different and not overlapping modes of action. Cumulative hematological toxicity seemed to be the only clinical limitation of this combinatorial approach. While LEN is known for its inhibition of cell-cycle progression, and promotion of apoptosis in cells that are not actively dividing, AZA’s effects depend on actively dividing cells [36]. The combination may maximize their additive effects, especially for patients with del(5q) [33]. 

We evaluated the combination of AZA and LEN in 20 del(5q) HR-MDS or AML patients (median age 69 years) within a phase I trial, including 35% of patients with AML [52]. In the trial, 15% of patients had an isolated del(5q), and 80% had del(5q) as part of a complex karyotype. Moreover, 65% of patients evaluable for molecular analysis had TP53 mutation at baseline. The combination strategy demonstrated a rate of 26% CR, and 42% CRi. Additionally, among previously untreated patients, hematologic and cytogenetic RR were 44% and 56%, respectively. Interestingly, we observed the disappearance of *TP53* mutations in patients achieving complete cytogenetic response and hematological remission after AZA and LEN, suggesting the potential benefit of combining both agents. This effect was not reported with LEN alone; therefore, whether the addition of AZA might improve treatment outcome remains to be determined in future studies [47,52]. 

However, the largest randomized study so far failed to show a marked benefit from this combination in HR-MDS patients. Sekeres et al. evaluated AZA alone versus a combination with LEN or vorinostat in HR-MDS and CMML, in a phase II study including 277 patients. Differences in ORR were not statistically significant, with 38% for patients receiving AZA, 49% for AZA plus LEN, and 27% for AZA plus vorinostat [53]. Furthermore, there was no survival benefit of a combination arm compared to AZA alone.

A phase II randomized Australian study (ALLG MDS4) also evaluated the regimen AZA ± LEN in 160 HR-MDS and AML patients with low blast count. AZA monotherapy showed an ORR of 56% versus 69% in the combination arm. Nevertheless, PFS demonstrated no benefit through the addition of LEN to AZA in this population [54].

Ades et al. evaluated escalating doses of LEN combined with ICT in a phase II study including 82 patients with HR-MDS or AML and 5q deletion. In the study, 46% of patients achieved CR, and the ORR was 58.5%. The study included 62 patients with complex karyotype, in which 44% achieved CR, median OS was 8.2 months [55]. 

### 3.3. Nucleoside Analog Sapacitabine

Sapacitabine is a novel oral nucleoside analog with a unique ability to induce single-strand DNA breaks after incorporation into DNA [56], and promises good efficacy with a favorable toxicity profile. The first encouraging results of sapacitabine have led to further combination trials evaluating this drug in combination with HMAs, such as DAC [57]. In AML cell lines, the active metabolite of sapacitabine, CNDAC, worked synergistic with HMA, and cells initially treated with HMA have been more apparent to CNDAC. Additionally, sapacitabine has recently shown sufficient activity when administered in alternating cycles with DAC. In a preceding Phase I/II study, eligible patients must have been ≥70 years with untreated AML, and unsuitable for, or unwilling to, receive standard ICT. In the trial, 46 elderly AML patients were treated with alternating cycles of DAC (20 mg/m^2^ iv five days of a 4-week cycle) and sapacitabine (300 mg orally twice daily three days/week of a 4-week cycle) [56]. Of the 46, 17 patients responded (37%) with 10 CRs, 2 PRs, and 5 major HIs. Median time to response was 2 cycles, and median OS was 238 days. A large, randomized phase III trial (SEAMLESS study) compared the regimen of sapacitabine administered in alternating cycles with DAC versus DAC monotherapy. The study did not show a significant improvement in OS as compared to DAC monotherapy in elderly AML patients [58].

### 3.4. Histonedeacetylaseinhibitors (HDACi) 

Transcriptional silencing of tumor-suppressor genes and other genes involved in differentiation and apoptosis is a result of DNA promotor hypermethylation and post-translational modification, like deacetylation of histone tails. Thus, inhibition of histone deacetylation and DNA hypermethylation can induce re-expression of silenced genes in leukemia in a synergistic fashion. In HR-MDS and AML, monotherapy with histonedeacetylaseinhibitors (HDACi) such as valproic acid, entinostat, vorinostat, panobinostat, or pracinostat has demonstrated only limited efficacy in phase I and II clinical trials [59,60]. Therefore, multiple clinical studies combining HMAs and HDACi are currently ongoing. One of the first and largest clinical studies combining both agents has been a phase II randomized clinical trial including 149 patients with MDS or AML, comparing AZA ± etinostat. The combination regimen has failed to demonstrate a significant improvement in RR or OS [61], possibly because HDACi are potent cell-cycle inhibitors, which may inhibit HMA incorporation into DNA. In this study, the combination of AZA and etinostat led to less demethylation compared to AZA monotherapy, suggesting pharmacodynamic antagonism. Preclinical and clinical data demonstrated that the specific doses and the sequence of use are very important in HMA–HDACi combinations, resulting in reduced synergism if both agents are not applied in an optimal sequence [61,62,63]. 

In another phase II study, 184 patients with HR-MDS or CMML were randomly assigned to AZA ± vorinostat. With a median follow-up of 23 months, the ORR was 38% for patients receiving AZA monotherapy, versus 27% for AZA plus vorinostat [53]. Additionally, 259 adults with AML or HR-MDS ineligible for ICT have been randomized to receive either AZA alone, or in combination with vorinostat. Again, the administration of vorinostat did not significantly increase RR (33% AZA versus 37% AZA + vorinostat), nor improve OS (1 year OS AZA 43% versus AZA + vorinostat 44%) [64].

In contrast, first data of the phase II study evaluating the combination regimen of pracinostat plus AZA in elderly patients with AML who were not eligible for induction chemotherapy are much more promising. For 50 patients who received the combination, CR, CRi and a morphologic leukemia free state was achieved in 42%, 4% and 6% of patients, respectively. The median duration of CR was 13.2 months, OS was 19.1 months after approximately 21 months of follow-up, and the estimated one year survival was 62% [65]. Thus, the combination AZA with pracinostat might be the first to improve OS compared to AZA monotherapy in AML patients; nevertheless, the only randomized phase III study is still running [66]. In contrast, a phase II study in HR-MDS patients did not demonstrate improved RR or improved survival rates (OS, EFS, PFS) following AZA plus pracinostat, compared with AZA monotherapy [67], leaving some uncertainties around this novel combination. In summary, the jury is still out on whether combining HDACi and HMA in older MDS and AML patients is of benefit.

### 3.5. Isocitrate Dehydrogenase (IDH) Inhibitors

IDH1- and IDH2-mutations are gain of function mutations, leading to the conversation of α-ketoglutarate to β-hydroxyglutarate (2-HG) [68]. 2-HG inhibits TET2, resulting in an impaired hydroxymethylation of DNA and in dysregulated methylation patterns [69]. In hematological disorders, mutations in IDH1 and IDH2 are occurring at a prevalence of 5–20% in *de novo* AML, and around 7.5% in *de novo* MDS patients [68]. Further studies identified IDH mutations, even occasionally among otherwise healthy older individuals with clonal hematopoiesis of indeterminate prognosis [70].

Enasidenib (AG-221) emerged as the first IDH mutation–specific inhibitor, and is a covalent inhibitor of IDH2 mutation [71]. In a phase I study, 40.3% of patients with relapsed/refractory AML responded to enasidenib monotherapy, including 19.3% achieving CR. Interestingly, IDH2 clearance was not required for response; patients with ≥6 co-mutations or NRAS co-mutations were less likely to attain a response [72].

Ivosidenib (AG-120) is one of several IDH1 inhibitors (IDH305, BAY-1436032, FT-2102) currently under development. In a phase I/II study exclusively enrolling IDH1-mutated patients with advanced hematologic malignancies, the ORR was 38.5%, with 17.9% achieving a CR. Moreover, one third of responding patients experienced elimination of IDH1 mutation [73]. In both enasidenib and ivosidenib, terminal differentiation of leukemic blasts has been observed, which may result in a differentiation syndrome (DS) in some patients. DS is a recognizable and potentially lethal clinical entity requiring prompt recognition and management [74].

Because IDH inhibitors target mechanisms in epigenetic regulation, potential synergistic effects with HMAs are assumed, and are currently under investigation [71].

### 3.6. Immune Checkpoint Inhibitors (ICI)

Under normal physiological conditions, immune checkpoints like CTLA-4, PD-1, and PD-L1 are crucial for the maintenance of self-tolerance to prevent autoimmunity, and to protect tissues from damage. In many tumors, the expression of checkpoint receptors is upregulated to evade immune attack. During an active anti-tumor immune response, checkpoint receptor expression can be increased in response to IFNγ, as a mechanism of adaptive immune resistance [75]. ICI targeting CTLA-4 and the PD-1/PD-L1 axis have shown impressive clinical activity in several types of solid tumors, including melanoma, non-small cell lung cancer, renal cell carcinoma, bladder cancer, head and neck squamous cell carcinoma, and Hodgkin lymphoma. In myeloid diseases, ICI have shown only modest clinical efficacy as single-agent therapies in clinical trials on advanced disease [76]. However, a recent report by our group about a patient with sAML undergoing single agent pembrolizumab (PD-1) treatment demonstrated a platelet response, together with clearance of IDH1 mutation [77]. Nevertheless, rationally designed combination approaches may be more effective than single-agent ICI in AML and MDS. It is known that HMAs can dampen immune response by upregulation of inhibitory immune checkpoint molecule expression, while enhancing anti-tumor immune response [78,79]. Up-regulation of PD-1, PD-L1, and PD-L2 in patients with AML and MDS during HMA therapy was associated with the emergence of resistance [79]. Thus, the combination of HMA and PD-1/PD-L1 inhibition may be a potential mechanism to prevent or overcome resistance to AZA or DAC. In a phase IB/II trial, the combination of nivolumab (PD-1) with AZA was evaluated in 53 relapsed or refractory AML patients who were not fit for ICT. The ORR was 34%, and 18% reached CR or CRi. Moreover, patients who achieved CR or CRi had higher total CD3^+^ and CD8^+^ T cell infiltrates in the BM prior therapy, and responders showed a progressive increase in BM CD8^+^ and CD4^+^ T cell infiltrates [52]. At a median follow-up of 6 months, only one out of 11 patients who achieved CR had lost response, suggesting a potentially more durable remission benefit among responders [79]. A study investigating dual combination of nivolumab (PD-1) and ipilimumab (CTLA-4) with AZA in relapsed or frontline elderly AML therapy has recently begun enrollment (NCT02397720). In intermediate-2/HR-MDS patients, a phase II study is currently evaluating AZA in combination with nivolumab, AZA with ipilimumab, and AZA with nivolumab and ipilimumab, as well as nivolumab, ipilimumab, or nivolumab monotherapy in patients who have failed prior therapy with HMAs (NCT02530463). In the study, 80% of patients in the AZA plus nivolumab arm showed a response including CR, mCR, or HI. On the other hand nivolumab monotherapy in HR-MDS showed no responses in all evaluable patients. Interestingly, monotherapy of ipilimumab demonstrated activity, with responses in 33% of HR-MDS patients after HMA therapy, suggesting that there might be a differential efficacy profile for PD-1 versus CTLA-4 inhibition in myeloid diseases [79]. In summary, combination approaches with HMAs plus ICI have shown early encouraging results in AML and MDS patients. Thus, ICI might be an attractive option for improving PFS, or to eliminate minimal residual disease post induction and consolidation in patients with HR-AML [80].

## 4. Conclusions

Compared to younger patients, older individuals with HR-MDS and AML have poor outcomes when conventional treatment strategies are used. Thus, there is a crucial need to improve frontline approaches for these patients. Presently, HMA leads to responses in less than half of patients, and all of them will eventually relapse. Due to dismal outcomes among older patients, new effective and tolerable treatment regimens improving survival and quality of life are needed. One of the most promising strategies currently under investigation is the combination of AZA or DAC with new investigational therapies, aiming to achieve long term synergistic activity and better patient outcomes. Approaches intended to prevent HMA resistance, e.g., by using checkpoint inhibitors, will determine the future of clinical research. Recent data also suggest a potential renaissance of intensive treatment strategies like CPX-351, a novel liposomal formulation of cytarabine and daunorubicin [90]. Participation in clinical trials is therefore an important prerequisite in the clinical care of older patients with HR-MDS and AML.

## Figures and Tables

**Table 1:** Selected phase I/II clinical trials examining the combination of HMA with new upcoming treatment strategies currently under investigation.

Agent	Trial Status	MDS/AML Subttyp	Efficacy
**HDACi + HMA**			
Vorinostat + AZA vs. AZA monotherapy	Phase II NCT00948064	Previously untreated AML or high-risk MDS	35% CR Vorinostat + AZA 44% CR AZA monotherapy Sekeres et al. 2017 [53]
Pracinostat + AZA	Phase II NCT01912274	Newly diagnosed AML	21% CR Garcia-Manero et al. 2017 [65]
Panobinostat + AZA	Phase I/IIb ACTRN12610000924055	Previously untreated AML or high-risk MDS	27.5% CR Garcia-Manero et al. 2017 [81]
**BCL-2 inhibitor + HMA**			
Venetoclax + AZA or DAC	Phase IB NCT02203773	Elderly, previously untreated AML	28% CR AZA + VEN 44% CR DAC + VEN DiNardo et al. 2018 [82]
**FLT3 inhibitors + HMA**			
Sorafenib + AZA	Phase II NCT01254890	Relapsed/refractory AML	16% CR Ravandi et al. 2013 [83]
Midostaurin + AZA	Phase I/II NCT01093573	Untreated or relapsed/refractory AML or high-risk MDS	18% CR Cooper et al. 2015 [84]
Quizartinib + LDAC/AZA	Phase I/II NCT01892371	Newly diagnosed/untreated or relapsed/refractory AML or high-risk MDS; FLT3-ITD positive	16,9% CR Swaminathan et al. 2017 [85]
**Cytotoxic Agents + HMA**			
Sapacitabine + DAC	Phase I/II NCT01211457	Newly diagnosed AML patients ≥70 years	16% CR Ravandi et al. 2011 [86]
**Cell Cyle Inhibitors + HMA**			
Rigosertib + AZA	Phase I/II NCT01926587	AML or high-risk MDS previously untreated or failed HMA	65% mCR Navada et al. 2017 [87]
**Isocitrate Dehydrogenase Inhibitors + HMA**			
Enasidenib or Ivosedinib + AZA	Phase I/II NCT02677922	Newly diagnosed, IDH1 or IDH2 mutation-positive AML	33% CR Enasidenib + AZA 42% CR Ivosidenib + AZA DiNardo et al. 2017 [88]
**Checkpoint Inhibitors + HMA**			
Ipilimumab/Nivolumab + AZA	Phase II NCT02530463	Frontline MDS, Relapsed/Refractory MDS	ongoing
Nivolumab + AZA	Phase II NCT02397720	Relapsed/Refractory AML >18 years, *de novo* AML ≥ 65 years	ongoing
Pembrolizumab + AZA	Phase II NCT02845297	Frontline AML ≥ 65 years, Relapsed/Refractory AML	ongoing
Durvalumab + AZA	Phase II NCT02775903	Frontline MDS, Frontline AML ≥ 65 years	ongoing
Atezolizumab + AZA	Phase I NCT02508870	Post-HMA failure MDS: Atezolizumab monotherapy vs. Atezolizumab + AZA Frontline MDS: Atezolizumab + AZA	ongoing
**Hedgehog Pathway Inhibitor + Standard Chemotherapy**			
Glasdegib + LDAC or DAC or cytarabine/daunorubicin	Phase Ib NCT01546038	Newly diagnosed AML or high-risk MDS	31% Cri Savona et al. 2018 [89]

Abbreviations: HDACi, Histone deacetylase inhibitors; AZA, Azacitidine; CR, complete remission; VEN, Venetoclax; DAC, Decitabine; FLT3, FMS-like tyrosine kinase-3; LDAC, Low-dose cytarabine; IDH, Isocitrate dehydrogenase.
